# Clinical Study of Correlation for the Intestinal and Pharyngeal Microbiota in the Premature Neonates

**DOI:** 10.3389/fped.2021.632573

**Published:** 2021-02-16

**Authors:** Sen Yang, Lina Qiao, Jing Shi, Liang Xie, Yang Liu, Ying Xiong, Hanmin Liu

**Affiliations:** ^1^Department of Pediatrics, West China Second University Hospital, Sichuan University, Chengdu, China; ^2^Key Laboratory of Birth Defects and Related Diseases of Women and Children (Sichuan University), Ministry of Education, West China Second University Hospital, Sichuan University, Chengdu, China

**Keywords:** intestinal micriobiota, pharyngeal microbiota, 16S rRNA sequencing, preterm neonate, gut-lung axis

## Abstract

**Objective:** There are mutual influences between intestine and lung, that propose a concept of the gut-lung axis, but the mechanism is still unclear. Microbial colonization in early life plays an important role in regulating intestinal and lung function. In order to explore the characteristics of early microbiota on the gut-lung axis, we studied the correlation between intestinal and pharyngeal microbiota on day 1 and day 28 after birth in premature neonates.

**Methods:** Thirteen neonates born at 26–32 weeks gestational age (GA) hospitalized at the neonatal intensive care unit (NICU) of the West China Second Hospital of Sichuan University were enrolled in this study. Stool samples and pharyngeal swabs samples were collected from each neonate on the first day (T1) and the 28th day (T28) after birth. Total bacterial DNA was extracted and sequenced using the Illumina MiSeq Sequencing System based on the V3–V4 hyper-variable regions of the 16S rRNA gene. Based on the sequencing results, the composition of the intestinal and pharyngeal microbiota was compared and analyzed.

**Results:** At T1, the difference in microbial composition between intestine and pharynx was not statistically significant. The intestinal microbiota was mainly composed of *Unidentified Enterobacteriaceae, Ralstonia, Streptococcus, Fusobacterium, Ureaplasma*, etc. The pharyngeal microbiota was mainly composed of *Ureaplasma, Bacteroides, Fusobacterium*, etc. *Ureaplasma* and *Fusobacterium* were detected in both intestine and pharynx. At T28, there was a significant difference in microbial composition between intestine and pharynx (*p* < 0.001). The intestinal microbiota was mainly composed of *Unidentified Clostridiales, Klebsiella, Unidentified Enterobacteriaceae, Enterobacter, Streptococcus*, etc. Pharyngeal microbiota was mainly composed of *Streptococcus, Rothia*, etc. *Streptococcus* was detected in both intestine and pharynx.

**Conclusions:** The intestine and pharynx of premature neonates have a unique microbial composition, and share some common microbiota. Whether these microbiotas play a role in the mechanism of gut-lung crosstalk needs further study.

## Introduction

Intestinal microbiota plays an important role in human health and disease (1). In recent years, numerous studies have shown that infant intestinal microbiota affects the growth and development of children (2, 3), and are associated with neonatal sepsis, neonatal necrotizing enterocolitis (4), childhood obesity (5), asthma, eczema (6), and diabetes (7), hypertension (8), and other diseases in adulthood. It is commonly believed that the uterus is sterile and the colonization of microbiota began after birth. But recent research results suggested that the intestinal microbiota could colonize in neonates before delivery. The concept of sterile uterus has been challenged, but the results are still controversial and worth further study (9, 10). The initial colonization of microbiota in the lung may affect the development of the respiratory system and even lead to disease (11, 12). Chronic respiratory diseases can affect the composition of the intestinal microbiota, and in turn, intestinal microbiota may affected the function of the respiratory system (13, 14). These results suggest that there is a crosstalk between the respiratory system and the intestine, which proposes a concept of the gut-lung axis (15). There are many studies on the gut-lung axis, but the mechanism is still unclear (16). Therefore, it is very crucial to study the microbial characteristics of the intestine and lung for further exploring the mechanism of the gut-lung axis.

The pharynx connects the oral cavity, nasal cavity, lower respiratory tract, and digestive tract. It communicates with the outside world and is exposed to a variety of exogenous and endogenous microbes. Thus, the pharynx is an ecological niche for potentially pathogenic microbe, which may cause local inflammation or lead to lung diseases (17). Previous studies have shown that there was a large amount of overlap between the pharyngeal microbiota and the respiratory microbiota (18), so the pharyngeal microbiota could, to some extent, reflect the characteristics of the respiratory microbiota. Besides, the pharyngeal swab is a fairly accurate method to define the composition of the respiratory microbiota. Therefore, studying the composition and characteristic of pharyngeal microbiota may open a window for exploring respiratory microbiota.

The concept of the gut-lung axis was born out of the observation that different lung diseases can be influenced by intestinal microenvironment changes and vice versa. The microbiota is an important factor responsible for interactions between these two sites in asthma (19). In addition, the intestinal microbiota of patients with severe bacterial pneumonia (20), cystic fibrosis (19), and influenza differs from that of healthy controls (21). Many studies show that early life is the most important period during which microbiota dysbiosis in the intestine may lead to the development of many respiratory diseases, as the intestine microbiota has a significant influence on immune cell maturation and resistance to pathogens (22).

In this study, we took premature neonates with GA <32 weeks as study subjects to analyze intestinal and pharyngeal microbial characteristics and influencing factors. Meanwhile, we studied the correlation between intestinal and pharyngeal microbiota, to provide some evidence for the role of early microbiota in the mechanism of the gut-lung axis.

## Patients and Methods

### Study Participants

Premature neonates who were delivered and hospitalized in the NICU of West China Second Hospital of Sichuan University from December 2019 to May 2020 were enrolled. Selection criteria were as follows: GA < 32 weeks; single birth; the birth weight of the infant appropriate for gestational age (between the 10–90th percentile of the average weight of the infant of the same gestational age) (23, 24). Exclusion criteria were as follows: infants with congenital malformations; intrauterine growth retardation; neonatal hypoxic-ischemic encephalopathy; immunodeficiency or severe infectious diseases; mother's antibiotic therapy for more than 3 days within 2 weeks before delivery; the neonate using antibiotics after birth more than 3 days within 2 weeks before delivery; the mother or the neonate used probiotics or prebiotics during perinatal period; and the guardian does not agree to participate or withdraws from the study. The following information was obtained from the medical records: duration of ruptured membranes; prenatal antibiotic use; delivery mode; GA; birth weight; gender. The study protocol was approved by the medical ethics committee of the West China Second Hospital of Sichuan University, and written informed consents were obtained from the parents or guardians of the neonates.

### Sample Collection Method

Stool samples were collected from baby diapers with sterile test tubes at T1 and T28. Pharyngeal secretions samples were collected with sterile pharyngeal swabs within 30 min after birth and T28. Stool and pharyngeal swabs samples were quickly placed in a −20°C refrigerator after being collected, and stored in a −80°C refrigerator within 24 h until further processing.

### DNA Extraction and Amplification

Total genome DNA from samples was extracted using Hexadecyltrimethy Ammonium Bromide (CTAB) method. DNA concentration and purity were detected by a spectrophotometer. According to the concentration, DNA was diluted to 1 ng/μl using sterile water. The bacteria genomic DNA was amplified with the 341F and 806R primers specific for the V3–V4 hypervariable regions of the 16S rDNA gene. All DNAs were amplified by following a protocol described previously (25). Samples were sequenced on an Illumina MiSeq platform according to the manufacturer's recommendations. The sequencing service was provided by Beijing Novogene Genomics Technology Co. Ltd (China).

### Data Processing

Paired-end reads from the original DNA fragments are merged by using FLASH (26), which is designed to merge paired-end reads when there are overlaps between reads 1 and reads 2. Paired-end reads were assigned to each sample according to the unique barcodes. Sequences were analyzed using QIIME software package (Quantitative Insights Into Microbial Ecology), and in-house Perl scripts were used to analyze alpha (within samples) and beta (among samples) diversity. First, reads were filtered by QIIME quality filters. Then we use pick_de_novo_otus. py to pick operational taxonomic units (OTUs) by making an OTU table. Sequences with ≥97% similarity were assigned to the same OTUs. We pick representative sequences for each OTU and use the RDP classifier (27) to annotate taxonomic information for each representative sequence. In order to compute Alpha Diversity, we rarify the OTU table and calculate the Shannon index and Chao1 estimator. QIIME calculates weighted unifrac, which are phylogenetic measures of beta diversity. We used weighted uniFrac for Principal Coordinate Analysis (PCoA). LefSe tool was implemented to identify differentially abundant taxa.

### Statistical Analysis

Continuous variables were reported as means ± standard deviations, and categorical data were presented as ratios or percentages. Non-parametric Wilcoxon rank-sum test was used to study differences in continuous variables and Fisher's exact tests were used to analyze categorical variables. *p* < 0.05 was considered statistically significant.

## Results

### Clinical Characteristics, and Influencing Factors of Microbial Colonization

In this study, 22 infants with GA <32 weeks in the NICU of West China Second Hospital of Sichuan University were included. Based on the exclusion criteria, 9 cases were excluded, and the remaining 13 cases were included in the final analysis (Table 1). At T1, 13 meconium samples were collected and included in the T1s group, and 13 pharyngeal swabs samples were collected and included in the T1y group. At T28, 13 stool samples were collected and included in the T28s group, and 13 pharyngeal swabs samples were collected and included in the T28y group. In total, 52 samples were collected from the enrolled neonates, 16S rRNA was amplified from 34 samples (6 samples in T1s group; 4 samples in T1y group; 13 samples in T28s group; 11 samples in T28y group; Table 2), and 2,944,688 reads were obtained. At T1, maternal antibiotics, GA, delivery mode, and gender had some effect on the microbial colonization of meconium and pharyngeal swabs, but these were not significant (Table 3).

**Table 1:** Clinical characteristics of neonates (*N* = 13).

**Subject**	Antibiotics, yes/no^*^	**GA, weeks**	**Delivery, cesarean/vaginal**	**Gender, male/female**	**Prolonged rupture of membranes, yes/no**
01	Yes	27.6	Cesarean	Female	Yes
02	Yes	27.6	Cesarean	Female	Yes
03	No	29.4	Vaginal	Male	No
04	No	28.1	Vaginal	Female	No
05	Yes	29.9	Vaginal	Male	Yes
06	Yes	29.0	Cesarean	Male	No
07	Yes	29.7	Cesarean	Male	Yes
08	Yes	28.7	Cesarean	Female	No
09	Yes	31.4	Cesarean	Female	No
10	No	31.9	Vaginal	Male	No
11	Yes	26.1	Vaginal	Male	Yes
12	Yes	27.9	Cesarean	Female	Yes
13	Yes	29.9	Vaginal	Male	Yes

* *Mother's intrapartum antibiotic therapy (duration < 3days)*.

**Table 2:** The result of 16S rRNA was amplified from samples (O: microbiota was detected).

**Subject**	**Meconium (T1s)**	**Pharyngeal swabs (T1y)**	**Stool (T28s)**	**Pharyngeal swabs (T28y)**
01	**O**		**O**	**O**
02		**O**	**O**	**O**
03	**O**		**O**	**O**
04	**O**	**O**	**O**	**O**
05		**O**	**O**	**O**
06			**O**	
07	**O**		**O**	**O**
08			**O**	**O**
09			**O**	**O**
10			**O**	**O**
11	**O**	**O**	**O**	**O**
12	**O**		**O**	
13			**O**	**O**

**Table 3:** Analysis of influencing factors of intestinal and pharyngeal microbial colonization.

**Factors**		**Microbiota was detected in meconium**	**Microbiota was detected in pharyngeal swabs**
Antibiotics	Yes (Total: 10)	4	3
	No (Total: 3)	2	1
	*P*-value	0.559	>0.999
Delivery	Vaginal (Total: 6)	3	3
	Cesarean (Total: 7)	3	1
	*P*-value	>0.999	0.266
GA	>28 w (Total: 9)	3	2
	<28 w (Total: 4)	3	2
	*P*-value	0.266	0.530
Gender	Male (Total: 7)	3	2
	Female (Total: 6)	3	2
	*P*-value	>0.999	>0.999

### Comparisons Between Intestinal and Pharyngeal Microbiota

**Figure 1:**
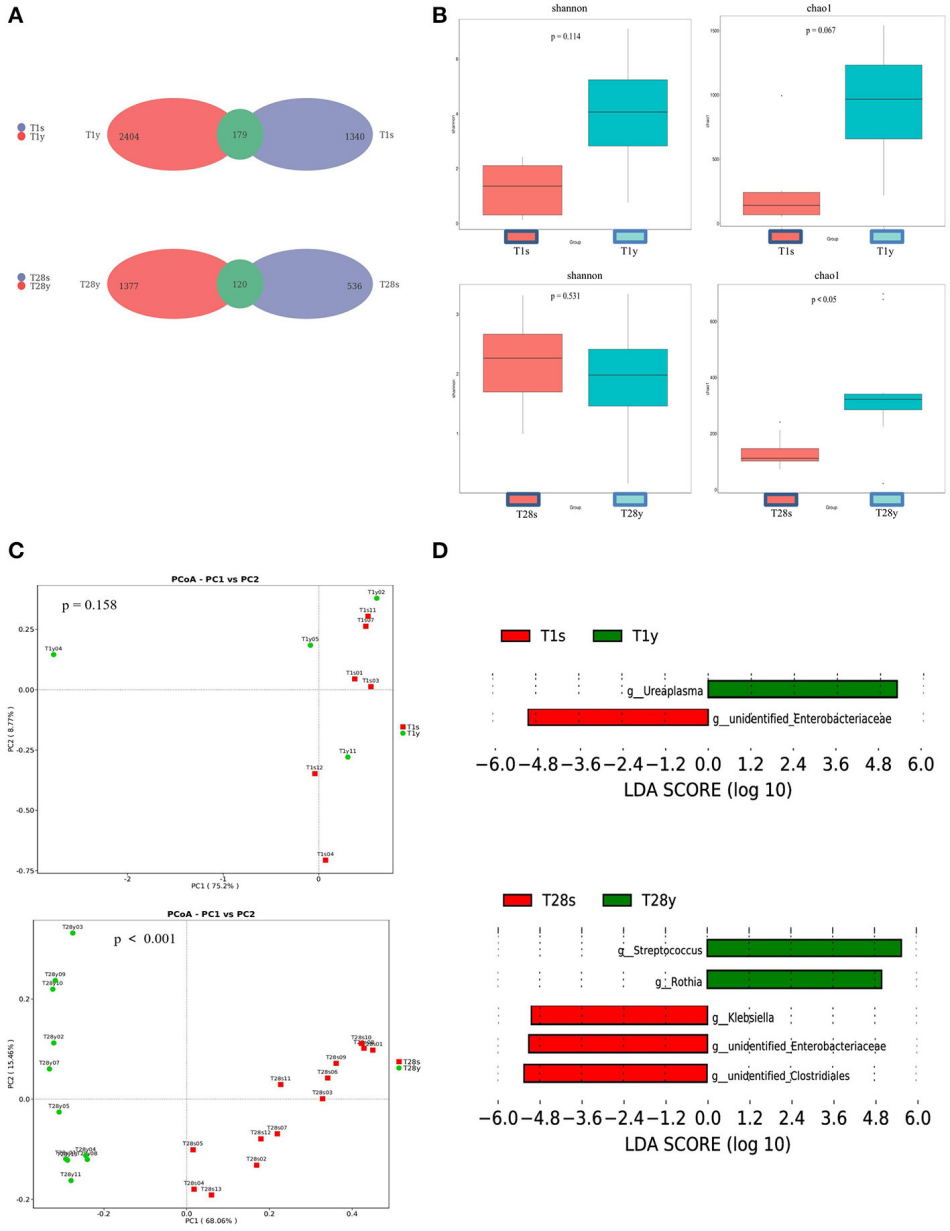
Comparisons between intestinal and pharyngeal microbiota. **(A)** OTUs were shared by the intestine and pharynx. **(B)** Comparison of the microbial biodiversity between intestine and pharynx, the shannon index, and chao1 were shown as estimators. **(C)** PCoA plot based on OTU abundance. Each point represents the intestinal or pharyngeal microbiota of a subject. **(D)** Histogram of the LDA scored for differentially abundant genera between intestine and pharynx. LDA scores were calculated by LDA effect size using linear discriminant analysis.

At T1, 1,340 OTUs were detected in the intestine, and 2,404 OTUs were detected in the pharynx, among which 179 OTUs were commonly shared. At T28, 536 OTUs were detected in the intestine, and 1,377 OTUs were detected in the pharynx, among which, 120 OTUs were commonly shared (Figure 1A). At T1, there was no significant difference in Shannon index and Chao1 index between the T1s group and the T1y group. At T28 there was no significant difference in Shannon index between the T28s group and the T28y group; the Chao1 index of the T28y group was higher than that of the T28s group, the difference was significant (*p* < 0.05; Figure 1B).

In order to compare the composition of the overall microbiota of the intestine and pharynx, PCoA was implemented based on the OTU level. At T1, the results of PCoA showed no difference in microbiota composition between the T1s group and the T1y group. At T28, the results of PCoA showed a significant difference in microbiota composition between the T28s group and the T28y group (*p* < 0.001; Figure 1C). Next, the LEfSe tool (28) was used to analyze microbiota in stool samples and pharyngeal swabs samples, and to detect potential significant differences in relative abundances between the intestinal and pharyngeal microbiota. There were significant differences in 2 genera between the T1s group and the T1y group. *Ureaplasma* was relatively more abundant in the T1y group, whereas *Unidentified Enterobacteriaceae* was relatively more abundant in the T1s group. There were significant differences in some genera between the T28s group and the T28y group. *Streptococcus, Rothia* were relatively more abundant in the T28y group, whereas *Unidentified Clostridiales, Unidentified Enterobacteriaceae, Klebsiella* were relatively more abundant in the T28s group (Figure 1D).

### The Microbial Composition of the Intestine and Pharynx at the Genus Level

**Figure 2:**
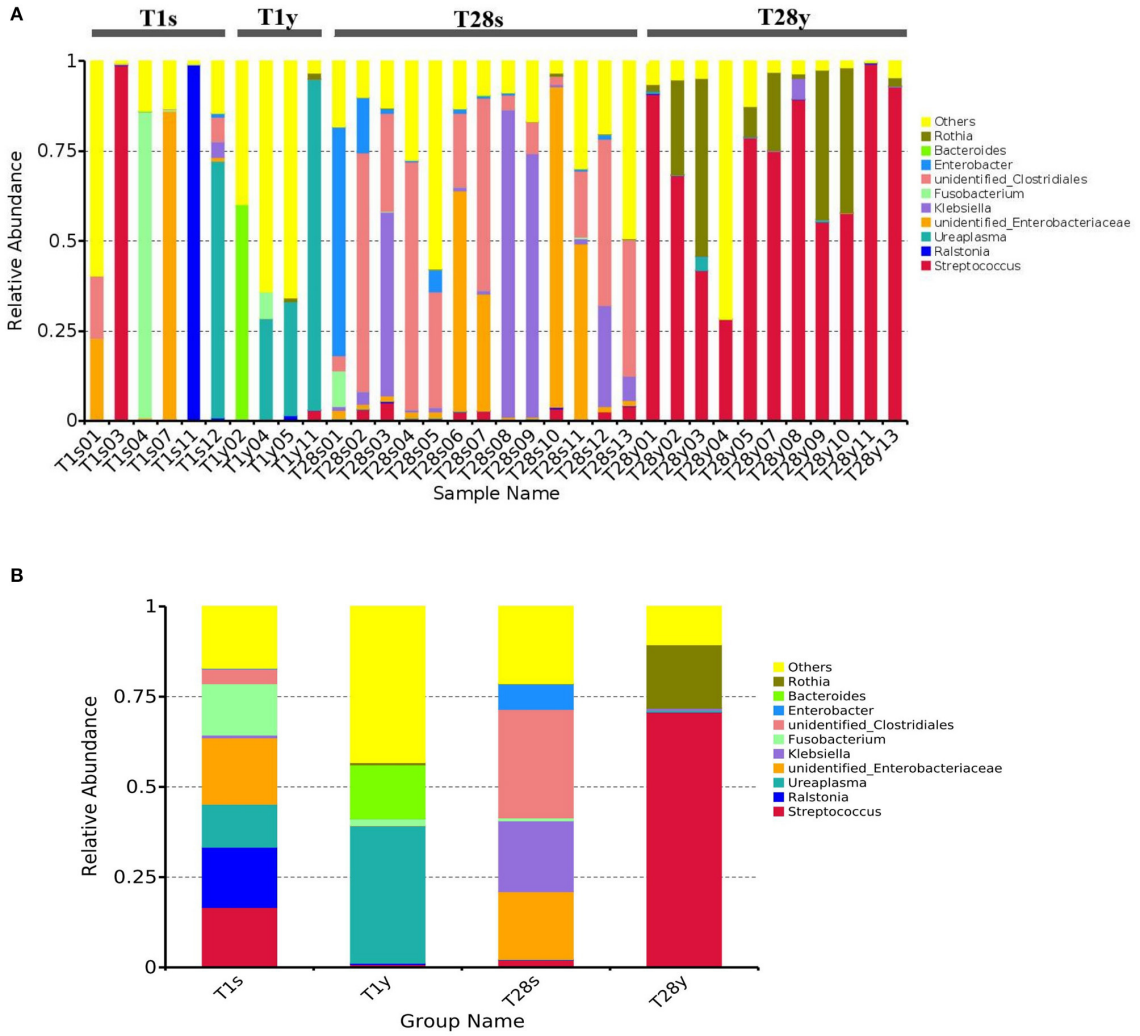
The composition of intestinal and pharyngeal microbiota.at the genus level. **(A)** The microbial composition of each sample. **(B)** Average microbiota of T1s, T1y, T28s, and T28y (T1s: meconium samples at T1; T1y: pharyngeal swabs samples at T1; T28s: stool samples at T28; T28y: pharyngeal swabs samples at T28).

We analyzed the microbial composition of the intestine and pharynx at the genus level and found that all samples were dominated by a specific genus (Figure 2A). At T1, intestinal microbiota was mainly composed of *Unidentified Enterobacteriaceae* (18.8%), *Ralstonia* (16.7%), *Streptococcus* (16.6%), *Fusobacterium* (14.2%), *Ureaplasma* (11.9%), etc. Pharyngeal microbiota was mainly composed of *Ureaplasma* (38.1%), *Bacteroides* (14.9%), *Fusobacterium* (1.8%), etc. At T28, intestinal microbiota was mainly composed of *Unidentified Clostridiales* (30.0%), *Klebsiella* (19.6%), *Unidentified Enterobacteriaceae* (18.8%), *Enterobacter* (7.0%), *Streptococcus* (2.0%), etc. Pharyngeal microbiota was mainly composed of *Streptococcus* (70.7%), *Rothia* (17.6%), etc. At T1, the relative abundance of *Ureaplasma* between the T1s group and the T1y group was 11.9 vs. 38.1% (*p* = 0.053); The relative abundance of *Unidentified Enterobacteriaceae* between the T1s group and the T1y group was 18.4 vs. 0.08% (*p* < 0.05). At T28, the relative abundance of *Streptococcus* between the T28s group and the T28y group was 2.0 vs. 70.7% (*p* < 0.05); the relative abundance of *Rothia* between the T28s group and the T28y group was 0.1 vs. 17.6% (*p* < 0.05); the relative abundance of *Unidentified Clostridiales* between the T28s group and the T28y group was 30.0 vs. 0.002% (*p* < 0.05); the relative abundance of *Unidentified Enterobacteriaceae* between the T28s group and the T28y group was 18.8 vs. 0.02% (*p* < 0.05); the relative abundance of *Klebsiella* between the T28s group and the T28y group was 19.6 vs. 0.5% (*p* < 0.05; Figure 2B).

## Discussion

Microbial colonization is a complex and dynamic process. In this study, premature neonates with GA <32 weeks were selected as study subjects. Microbiota was detected in 6 meconium samples and 4 pharyngeal swabs samples at birth, which seems to be a challenge to the concept of a sterile uterus, but it needs to be determined by a larger sample size and the exclusion of more interfering factors. Maternal antibiotics, GA, delivery mode, and gender had no significant effect on the microbial detection rate of meconium and pharyngeal swabs, this is inconsistent with the results of some previous studies that the delivery mode affects the microbiota colonization of the neonates (29). The possible reason is that our sample collection was carried out immediately after birth, and the colonization of environmental microbiota has not been completed.

Through Venn diagram analysis, at T1, intestine and pharynx shared more OTUs than at T2. We may speculate that this pattern of colonization can be driven by the developing host immunity (30), or, alternatively, one pioneer group of bacteria (31) might metabolically prime the environment for replacement by a particular successor. Our observations support the hypothesis that temporal factors influence the microbial colonization of preterm infants, such as there was no significant difference in Shannon index between the T28s group and the T28y group. The Chao1 index of the T28y group was higher than that of the T28s group, and the difference was significant. The results show that the pharyngeal microbiota diversity was higher than that of the intestinal and that the low abundance and rare species are present in the community at T28y group. Through PCoA analysis, there was no significant difference in microbial composition between the T1s group and the T1y group. At T28, there was a significant difference of microbial composition between the T28s group and the T28y group. *Streptococcus, Rothia* were relatively more abundant in the T28y group, whereas *Unidentified Clostridiales, Klebsiella, Unidentified Enterobacteriaceae, Enterobacter* were relatively more abundant in the T28s group.

The analysis of microbial composition show that, at T1, the intestine was dominated by *Unidentified Enterobacteriaceae, Ralstonia, Streptococcus*, etc. among which *Unidentified Enterobacteriaceae* and *Streptococcus* are potential pathogenic microbes, and the rest are mostly pathogenic microbes. It suggests that premature neonates have very few beneficial microbes in the intestine, but a large number of potentially pathogenic microbes and pathogenic microbes, which is consistent with previous studies (32). At T1, pharyngeal microbiota was mainly composed of *Ureaplasma, Bacteroides, Fusobacterium*, etc, which are similar to the oropharyngeal microbiota of infants (33, 34). *Ureaplasma* was detected in both intestine and pharynx, the relative abundance has no significant difference between the T1s group and the T1y group. This may indicate that the origin of intestinal and pharyngeal microbiota may be the same at birth. The relative abundance and composition of the intestinal and pharyngeal microbiota was significantly different on the 28th day after birth. *Streptococcus* mainly existed in the pharynx, while *Unidentified Clostridiales* mainly existed in the intestine. We analyzed the characteristics of intestinal microbiota at different time, the results show that the intestinal microbiota colonization with beneficial bacteria is delayed in preterm infants, while the number of potentially pathogenic bacteria is high. *Ureaplasma* was relatively more abundant in the T1y group, *Streptococcus* was relatively more abundant in the T28y group. There was significant difference between early and late pharyngeal microbiota. These preliminary and explorative results suggested that the neonatal microbial colonization is a highly dynamic process, which is sensitive to environmental factors.

However, several potential limitations should be taken into consideration. Firstly the sample amount might not be adequate. Secondly, if the characteristics of neonatal microbiota and maternal microbiota were studied together, it is more meaningful to know the initial source of microbial colonization.

In conclusion, the study's results suggest that, in some neonates, the microbiota may exist in both the intestine and the pharynx at birth. There was no significant difference between intestinal and pharyngeal microbiota composition on day 1, but on day 28, site-specific microbial was already established. Despite the initial similarity of pharyngeal and gut microbiotas, the specific bacterial genera were determined in both sites. Further we hypothesize that these genera may contribute to the functioning of the gut-lung axis.

## Data Availability Statement

The original contributions presented in the study are included in the article/supplementary material, further inquiries can be directed to the corresponding author/s. https://figshare.com/s/81253de749d49bcb0898.

## Ethics Statement

The studies involving human participants were reviewed and approved by Ethics Committee of West China Second University Hospital, Sichuan University. Written informed consent to participate in this study was provided by the participants' legal guardian/next of kin.

## Author Contributions

SY: data collection, management and analysis, and manuscript drafting and revision. LQ and JS: data collection and manuscript revision. LX: site oversight and manuscript revision. YX and HL: study concept and design, interpretation of results, manuscript revision, and final approval. All authors contributed to the article and approved the submitted version.

## Conflict of Interest

The authors declare that the research was conducted in the absence of any commercial or financial relationships that could be construed as a potential conflict of interest.
